# Subproteome of *Lachesis muta rhombeata* venom and preliminary studies on LmrSP-4, a novel snake venom serine proteinase

**DOI:** 10.1590/1678-9199-JVATITD-1470-18

**Published:** 2019-04-15

**Authors:** Gisele A Wiezel, Karla CF Bordon, Ronivaldo R Silva, Mário SR Gomes, Hamilton Cabral, Veridiana M Rodrigues, Beatrix Ueberheide, Eliane C Arantes

**Affiliations:** 1 Department of Physics and Chemistry, School of Pharmaceutical Sciences of Ribeirão Preto, University of São Paulo, Av. do Café s/n, 14040-903, Ribeirão Preto, SP, Brazil.; 2 Institute of Biosciences, Letters and Exact Sciences, Universidade Estadual Paulista, Rua Cristóvão Colombo, 2265, 15054-000, São José do Rio Preto, SP, Brazil.; 3 Institute of Genetics and Biochemistry, Federal University of Uberlândia, Av. Pará, 1720, 38400-902, Uberlândia, MG, Brazil.; 4 Department of Chemical and Physical, State University of Southwest Bahia, Rua José Moreira Sobrinho, até 873 874, 45506-210, Jequié, BA, Brazil.; 5 Department of Pharmaceutical Sciences, School of Pharmaceutical Sciences of Ribeirão Preto, University of São Paulo, Av. do Café s/n, 14040-903, Ribeirão Preto, SP, Brazil.; 6 Proteomics Resource Center, New York University Langone Medical Center, 430 East 29th St., 10016, New York City, USA.

**Keywords:** bushmaster, snake venom, SVSP, kallikrein-like, plasminogen activator, kininogenase, lectin, protease, envenomation

## Abstract

**Background:**

*Lachesis muta rhombeata* is one of the venomous snakes of medical importance in Brazil whose envenoming is characterized by local and systemic effects which may produce even shock and death. Its venom is mainly comprised of serine and metalloproteinases, phospholipases A_2_ and bradykinin-potentiating peptides. Based on a previously reported fractionation of *L. m. rhombeata* venom (LmrV), we decided to perform a subproteome analysis of its major fraction and investigated a novel component present in this venom.

**Methods:**

LmrV was fractionated through molecular exclusion chromatography and the main fraction (S5) was submitted to fibrinogenolytic activity assay and fractionated by reversed-phase chromatography. The N-terminal sequences of the subfractions eluted from reversed-phase chromatography were determined by automated Edman degradation. Enzyme activity of LmrSP-4 was evaluated upon chromogenic substrates for thrombin (S-2238), plasma kallikrein (S-2302), plasmin and streptokinase-activated plasminogen (S-2251) and Factor Xa (S-2222) and upon fibrinogen. All assays were carried out in the presence or absence of possible inhibitors. The fluorescence resonance energy transfer substrate Abz-KLRSSKQ-EDDnp was used to determine the optimal conditions for LmrSP-4 activity. Molecular mass of LmrSP-4 was determined by MALDI-TOF and digested peptides after trypsin and Glu-C treatments were analyzed by high resolution MS/MS using different fragmentation modes.

**Results:**

Fraction S5 showed strong proteolytic activity upon fibrinogen. Its fractionation by reversed-phase chromatography gave rise to 6 main fractions (S5C1-S5C6). S5C1-S5C5 fractions correspond to serine proteinases whereas S5C6 represents a C-type lectin. S5C4 (named LmrSP-4) had its N-terminal determined by Edman degradation up to the 53^rd^ amino acid residue and was chosen for characterization studies. LmrSP-4 is a fibrinogenolytic serine proteinase with high activity against S-2302, being inhibited by PMSF and benzamidine, but not by 1,10-phenantroline. In addition, this enzyme exhibited maximum activity within the pH range from neutral to basic and between 40 and 50 °C. About 68% of the LmrSP-4 primary structure was covered, and its molecular mass is 28,190 Da.

**Conclusions:**

Novel serine proteinase isoforms and a lectin were identified in LmrV. Additionally, a kallikrein-like serine proteinase that might be useful as molecular tool for investigating bradykinin-involving process was isolated and partially characterized.

## Background

The genus *Lachesis* is represented only by the species *L. muta* in Brazil and the ophidian accidents caused by these snakes are the second most lethal (mortality/number of accidents) in this country [1, 2]. This lethality index might be derived from the high amount of venom injected into the victim but also to the lacking (or delayed) treatment access in remote regions [3-5]. Ophidian accidents caused by *Lachesis* are very significant, especially due to the quantity of venom that this snake is able to inject into the victim. The envenoming caused by this snake is characterized by local pain, edema, hemorrhage, necrosis, nausea, vomiting, coagulopathies (e. g. hypofibrinogenemia), renal disturbs, bradycardia, hypotension and shock, followed by a fast and irreversible hypotension which leads to death [2, 3, 6-11].

*Lachesis muta rhombeata* venom (LmrV) comprises mainly bradykinin potentiating peptides (BPPs), serine proteinases, metalloproteinases and phospholipases A_2_ (PLA_2_) besides lectins, cysteine-rich secretory proteins (CRISP), L-amino acid oxidase (LAAO), vascular endothelial growth factor (VEGF) and phospholipase B [12, 13]. As described above, *Lachesis* venoms present local and systemic alterations. The serine proteinases from these venoms are usually involved in hydrolysis of coagulation factors and may act in blood pressure reduction [14-16] whereas metalloproteinases present hemorrhagic actions [17, 18]. On the other hand, PLA_2_s are myotoxic enzymes that cause extensive local damage in the bite site [19, 20] and LAAO present high cytotoxic action [21].

However, there is a certain difficulty in maintain *Lachesis* snakes in captivity due to the peculiar characteristics of their natural habitat [22] and this hinders the study of their venoms. Few components have been isolated from LmrV, including LAAO [21], PLA_2_ [19], serine proteinases [23-25] and hyaluronidase [13]. Hyaluronidase was the last isolated component to be reported about 3 years ago. Recently, the *in vitro* and *in vivo* effects of a synthetic version of a BPP identified in this venom was reported [26].

Studying animal venoms and their components have helped, for example, to understand the envenoming process [27], improve the effectiveness of antivenoms [28], search for targets in the antivenom therapy [29] and to develop diagnostic reagents [30] and more specific therapeutic agents to fight against different diseases [31, 32]. Therefore, based on a previous fractionation of LmrV [13], we decided to investigate the subproteome of its major fraction (S5) and search for compounds which may be further investigated as a tool in the development of novel drugs or diagnostic reagents.

## Methods

### Venom

*L. m. rhombeata* (IBAMA registration number 647.998) was maintained by the Serpentarium “Bosque da Saúde”, city of Americana (São Paulo state, Brazil, 22º 44' 21" S, 47º 19' 53" W). The venom provided was desiccated and stored at -20 ºC until used.

### Venom fractionation protocol

LmrV (23 mg) was dissolved in 500 μL of 50 mM sodium acetate buffer with 0.15 M NaCl (pH 6) and centrifuged (13,400 *xg*, 4 ºC, 10 min). The supernatant was applied on a *HiPrep Sephacryl S-100 HR* column (1.6 x 60 cm; GE Healthcare, Sweden) previously equilibrated with the same buffer, and fractions of 1.5 mL were collected at a flow rate of 0.5 mL/min [13]. The S5 fraction was pooled and submitted to a reversed-phase fast protein liquid chromatography (RP-FPLC) on a 214MS^®^ C4 column (250 x 4.6 mm, 5 µm, 300 Å, Grace™ Vydac™, USA) previously equilibrated with 0.1% trifluoroacetic acid (TFA). Samples were eluted following a discontinuous gradient of 60% acetonitrile (ACN) in 0.1% TFA at 0.7 mL/min. Absorbance was monitored at 280 nm by FPLC Äkta Purifier UPC-10 system (GE Healthcare) in both steps. Protein recovery of the eluted fractions was calculated by the software Unicorn^®^ 5.20 (GE Healthcare).

### N-terminal sequencing

The N-terminal sequences of the fractions eluted from the RP-FPLC were determined by Edman degradation [33]. About 200 pmol of each fraction S5C1-S5C5 and 400 pmol of fraction S5C6 were sequenced by an automated protein sequencer model PPSQ-33A (Shimadzu Co., Japan) according to the manufacturer’s instructions. The obtained sequences were compared with non-redundant protein sequences (nr) from snakes (taxid: 8570) available at BLAST database using the online blastp suite (protein-protein blast) (http://blast.ncbi.nlm.nih.gov/Blast.cgi).

### Internal peptides analysis

The fraction S5C4 (20 µg) dissolved in NuPAGE LDS sample buffer (Life Technologies, USA) was reduced with 0.2 M 1,4-dithiothreitol (DTT) and alkylated with 0.5 M iodoacetamide. Reduced and alkylated sample was divided into two wells to be loaded in a NuPAGE 4-12% Bis-Tris Gel (Novex, USA) and gel run was performed at 200 V for 45 min using NuPAGE^®^ MOPS-SDS Running Buffer (Invitrogen). Gel was stained with GelCode^®^ Blue Stain Reagent (Thermo Scientific, USA) and SeeBlue^®^ Plus2 Pre-stained Protein Standard (Invitrogen, USA) was used as molecular weight marker (4-250 kDa).

Protein band from each well was excised from the gel, destained and digested with 200 ng of modified trypsin (Promega, USA) or 400 ng of endoproteinase Glu-C (Roche, Germany) at 25 °C, for 15 h and under shaking. Peptides were extracted using C_18_ ZipTip ZTC18S960 (Merck Millipore, USA) [34].

1/5 of each digested sample was loaded onto an EASY-Spray PepSwift Monolithic Capillary column (Thermo Scientific) using an Easy-nLC 1000 (Thermo Scientific) coupled to an Orbitrap Elite™ Mass Spectrometer (Thermo Scientific). Peptides were eluted for 65 min using a gradient from 2 to 90% of ACN in 0.5% acetic acid. High-resolution full MS spectra were acquired with resolution of 60,000 (at m/z 400) and automatic gain control (AGC) target of 1e6. The twenty most intense ions were subsequently fragmented by higher-energy collisional dissociation (HCD) in a data-dependent mode. The HCD MS/MS spectra were acquired with a resolution of 15,000 (at m/z 400), AGC target of 5e4, normalized collision energy of 27, and isolation window of ±2 Da. Additionally, another run was performed and here the 20 most intense ions were fragmented by electron transfer dissociation (ETD) also in a data-dependent mode and the ETD MS/MS spectra were acquired with a resolution of 15,000 (at m/z 400), AGC target of 5e4, activation time of 60 ms, and isolation window of ±2 Da after each full MS scan.

Data were searched against a database downloaded from UniProt [35] using the keywords “serine proteinase” and “*Lachesis*”. This database was downloaded in July 13^th^, 2015 and contains the 5 sequences of serine proteinases available for this snake genus. The search was performed by the error tolerant search engine Byonic™ v2.3.5 (Protein Metrics, USA) [36], setting the protein false discovery rate (FDR) cutoff as 1%, the precursor tolerance as 10 ppm and the fragment tolerance as 20 ppm and the ‘wildcard’ feature (which adds customizable mass windows to specific or all amino acids to search amino acid substitutions/modifications) as ±150 Da. Cysteine residues are carbamidomethylated and methionine oxidation, pyro-Glu at N-terminal, amidated C-terminal and HexNAc addition were set as variable modifications. Results were manually confirmed by *de novo* sequencing to exclude false positives.

### Carbohydrate content analysis

MS/MS data were searched as described in the section above and both the ‘wildcard’ feature (adding a customizable mass window of +5000 Da to specific or all amino acids) and HexNAc (+203.079373 Da) at N-Glycan set as variable modification were enabled in Byonic to detect possible glycosylation sites in LmrSP-4 amino acid sequence in comparison to the sequences available in the database.

In addition, LmrSP-4 (~50 μg) diluted in 50 mM ammonium bicarbonate buffer (AMBIC) was added to 5% SDS (0.36%, final concentration) and 1 M DTT (71 mM, final concentration) and heated at 95 °C for 10 min. Reduced LmrSP-4 was let at room temperature for 5 min and added of 10% Triton™ X-100 (1.25%, final concentration). Deglycosylation reaction occurred with 10 units of PNGase F (G5166, Sigma-Aldrich, USA) at 37 °C for 5 h. Reaction was stopped by heat denaturation (100 °C, 10 min) and sample was stored at -20 °C until used. Positive control for glycosylation was the reduced LmrSP-4 with 5% SDS, 1 M DTT and 10% Triton™ X-100 as described above. Reduced and deglycosylated LmrSP-4 were analyzed by 13.5% SDS-PAGE [37] and gel was stained with periodic acid-Schiff to the detection of glycoproteins [38] and *Coomassie Brilliant Blue G-250* ^®^ (Sigma-Aldrich).

### *In silico* analysis of the amino acid sequences

Multiple sequence alignments were generated by Clustal Omega [39] and edited by the free software BioEdit (http://www.mbio.ncsu.edu/bioedit/bioedit.html) and the ESPript server [40].

### Molecular mass

LmrSP-4 sample was dissolved in water and diluted in the proportion 1:1 with sinapinic acid (5 mg/mL) in 50% ACN and 0.1% TFA, spotted onto a sample plate and allowed to dry at room temperature. LmrSP-4 molecular mass was determined by matrix-assisted laser desorption/ionization time-of-flight mass-spectrometry (MALDI-TOF MS) using the system Ultraflex II MALDI TOF/TOF (Bruker Daltonics, USA). The equipment was calibrated in the masse range 5.7-66.4 kDa using the kit ProteoMass™ Protein MALDI-MS Calibration Kit (Sigma-Aldrich), and it was operated in linear positive mode with 4400 shots per spectrum. Data was analyzed by the software flexAnalysis version 3.3 (Bruker Daltonics).

### Fibrinogenolytic activity assay

Fibrinogenolytic activity assay was performed according to modifications on Edgar and Prentice method [41]. Bovine fibrinogen (0.5 mg/mL; Sigma Chemical Co.) in 0.1 M Tris-HCl buffer (pH 8) was incubated with S5 or LmrSP-4 at 37 ºC for 3 h. Reaction was stopped with 50 mM Tris-HCl buffer (pH 6.8) containing 10% glycerol, 10% β-mercaptoethanol, 2% SDS and 0.05% bromophenol blue. Sample was taken under boiling for 5 min and analyzed by 12% SDS-PAGE [37]. Gel was stained with *Coomassie Brilliant Blue G-250* ^®^. The action of possible inhibitors was evaluated pre-incubating the enzyme with ethylenediamine tetraacetic acid (EDTA, 20 mM final concentration) and phenylmethylsulfonyl fluoride (PMSF, 10 mM final concentration) at 37 °C for 15 minutes.

### Enzyme activity evaluation upon chromogenic substrates

Amidolytic activities upon chromogenic substrates (S-2238: substrate for thrombin; S-2302: substrate for plasma kallikrein; S-2251: substrate for plasmin and streptokinase-activated plasminogen; and S-2222: substrate for Factor Xa) were performed as described in the manufacturer’s protocols (Chromogenix, Italy). Each substrate was dispersed in ultrapure water (18.2 MΩ cm, Milli-Q water, Millipore, USA) and diluted in 50 mM Tris-HCl buffer with 5 mM CaCl_2_ (pH 7.5). The substrate solution and LmrSP-4 (1 μg dispersed in the same buffer) were added to a 96-well microplate and incubated at 37 ºC for 40 min. After that, the samples absorbance was measured at 405 nm. The assay was performed in triplicate and the negative control consisted of chromogenic substrate solution added to the previous buffer. Enzyme activity was also evaluated in the presence of some proteinase inhibitors (benzamidine, 1,10-phenantroline and PMSF). For this purpose, the enzyme (1 μg) was previously incubated with each inhibitor (20 mM) at 37 ºC for 60 min.

### Optimal conditions

Optimal conditions (pH and temperature) for LmrSP-4 were determined with the fluorescence resonance energy transfer (FRET) Abz-KLRSSKQ-EDDnp substrate as previously reported [42]. Proteolytic activity was monitored using excitation and emission wavelengths of 320 and 420 nm, respectively, in a Lumina Fluorescence Spectrometer (Thermo Scientific). Effects of pH were evaluated using the following 0.1 M buffer solutions: Mes (pH 6.0 and 6.5), Hepes (pH 7.0, 7.5 and 8.0) and Bicine (pH 8.5 and 9.0) at 40 °C. Temperature effects were evaluated in 0.1 M Hepes buffer (pH 7) in the range 40-60 °C.

### Statistical analysis

Statistical analyses for the LmrSP-4 activity upon chromogenic substrates were performed by GraphPad Prism 6.01 (GraphPad Software Inc., USA) using unpaired t-test and comparing the activity in each tested substrate with its own negative control. The data (mean ± standard error of the mean) obtained in the activity assays against chromogenic and FRET substrates were analyzed by one-way ANOVA followed by Dunnett’s multiple comparisons test and considered statistically significant when *p* < 0.05.

## Results

### Venom fractionation and N-terminal sequencing

**Figure 1.**
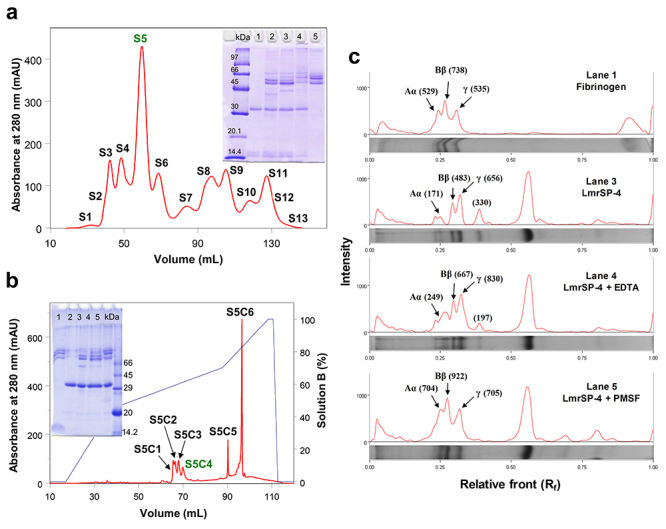
*Lachesis muta rhombeata* venom fractionation. **A:** LmrV was applied on a *HiPrep Sephacryl S-100 HR* previously equilibrated with 50 mM sodium acetate buffer with 0.15 M NaCl (pH 6) and fractions were eluted in the same buffer. Insert: 13.5% SDS-PAGE of S5 submitted to fibrinogenolytic activity. Gel run was carried out at 90 V. Lanes: kDa: molecular weight marker; 1: S5; 2: S5 plus fibrinogen; 3: S5 plus fibrinogen and EDTA; 4: S5 plus fibrinogen and PMSF; 5: fibrinogen (negative control). **B:** Fraction S5 was submitted to RP-FPLC on a C4 column previously equilibrated with 0.1% TFA (solution A). Fractions S5C1-S5C6 were eluted using a gradient of 60% ACN in 0.1% TFA (solution B). Insert: 13.5% SDS-PAGE of S5C4 submitted to fibrinogenolytic activity. Gel run was carried out at 90 V. Lanes: 1: fibrinogen (negative control); 2: S5; 3: S5 plus fibrinogen; 4: S5 plus fibrinogen and EDTA; 5: S5 plus fibrinogen and PMSF; kDa: molecular mass marker. **C:** Densitometry analysis of the protein bands from the SDS-PAGE of S5C4 submitted to fibrinogenolytic activity. After dried, the gel was scanned and analyzed by the system Gel Doc™ EZ Gel Documentation and the software Image Lab™ (Bio-Rad, USA). The intensities of the main bands are in parentheses.

LmrV was fractionated by molecular exclusion chromatography (Fig. 1a) and the majoritarian fraction (S5), which corresponds to approximately 26% of the crude soluble LmrV (Table 1), presented proteolytic activity upon fibrinogen except in the presence of PMSF (Fig. 1a). S5 fraction was then fractionated by RP-FPLC, giving rise to 6 main subfractions named S5C1-S5C6 (Fig. 1b). These fractions were submitted to N-terminal sequencing and results are shown in Table 2. Fractions S5C1-S5C5 were identified as belonging to the snake venom serine proteinase (SVSP) family and represent together more than 50% of the protein families identified in the fraction S5. However, S5C5 fraction also contains lectin. The S5C6 belongs to the lectins family and accounts to about 42% of the S5 proteins (Table 2). A complete list containing the blast results for each fraction is available in Additional file 1.

**Table 1:** Recovery of fractions in LmrV fractionation protocol

Fractionation step	Sample	Protein (mg)	Yield (%)
Dispersion of venom in the buffer	LmrV	13.25	100
Molecular exclusion chromatography	S5	3.49	26.3
Reversed-phase chromatography
	S5C1	0.33	2.52
	S5C2	0.36	2.69
	S5C3	0.49	3.68
	S5C4	0.50	3.78
	S5C5	0.34	2.57
	S5C6	1.46	11.06

**Table 2.** N-terminal assignment of the main subfractions from RP-FPLC

Fraction	Proportion in S5 (%)	N-terminal sequence	Accession number^*^	Protein name	Protein family
S5C1	9.6	VIGGDECNINEHRFLVALYDPDGFFCGGTL	C0HLA1	LmrSP-2	svSP
S5C2	10.22	IVGGDECNINEHRFLVALYDPDGFFC	C0HLA2	LmrSP-3	svSP
S5C3	14.00	IVGGDECNINEHRFLVALYDPDGFFC	-	-	svSP
S5C4	14.37	VFGGDECNINEHRSLVVLFDSDGFLCAGTLINKEWVLTAAHCDSENFQMQLGV	C0HLA3	LmrSP-4	svSP
S5C5	9.76	VFGGDECNINEHRSLVVLFDSDGFL	-	LmrSP-5	svSP
		DCPSGWSSYEGHCYR	-	-	lectin
S5C6	42.05	DCPSGWSSYEGHCYRVFNEPKNWADAERFCKLQPKHSHLV	C0HLA4	LmrLEC-1	lectin

^*^ Sequences will be available for public access after the article publication

### LmrSP-4 structural characterization

**Figure 2.**
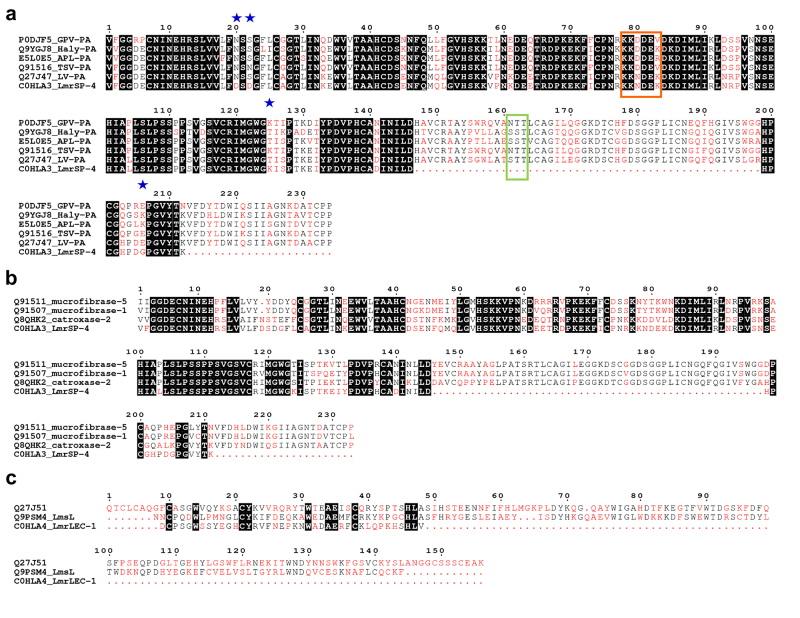
Multiple sequence alignments of SVSPs and snake venom lectins. Multiple sequence alignment among **(a)** LmrSP-4 and plasminogen activators SVSPs, **(b)** LmrSP-4 and kallikrein-like SVSPs and **(c)** LmrLEC-1 and lectins from *Lachesis*. The highly conserved residues are highlighted in black while low consensus residues are shown in red. Main differences between LmrSP-4 and LV-PA discussed in the text are indicated by blue stars. The green box shows a region with a putative N-glycosylation site in LmrSP-4 and the orange box represents the peptide important for the interaction between TSV-PA and its substrate plasminogen. P0DJF5: venom plasminogen activator GPV-PA from *Trimeresurus albolabris*; Q9YGJ8: venom plasminogen activator Haly-PA from *Gloydius brevicaudus*; E5L0E5: venom plasminogen activator APL-PA from *Agkistrodon piscivorus leucostoma*; Q91516: venom plasminogen activator TSV-PA from *Trimeresurus stejnegeri*; Q27J47: venom plasminogen activator LV-PA from *L. m. muta*; C0HLA3: serine proteinase LmrSP-4 from *L. m. rhombeata*; Q91511: beta-fibrinogenase mucrofibrase-5 from *Protobothrops mucrosquamatus*; Q91507: beta-fibrinogenase mucrofibrase-1 from *Protobothrops mucrosquamatus*; Q8QHK2: SVSP catroxase-2 from *Crotalus atrox*; Q27J51: C-type lectin from *L. muta*; Q9PSM4: C-type lectin from *L. stenophrys*.

**Figure 3.**
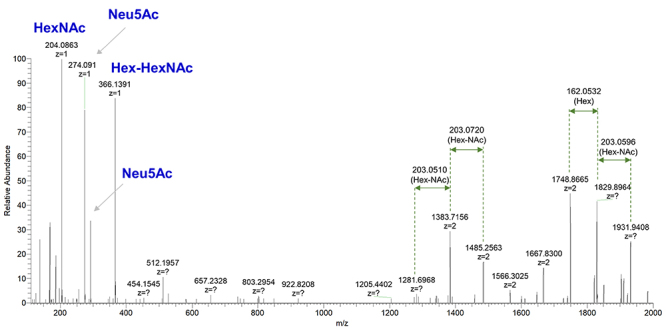
Carbohydrate content analysis by MS/MS. HCD MS/MS spectrum of the ion [M+4H]^4+^ = 4971.2538 from a LmrSP-4 glycopeptide acquired by an Orbitrap Elite™ Mass Spectrometer with resolution of 60,000 (at m/z 400). Abbreviations: HexNAc: N-acetylhexoseamine; Hex-HexNAc (hexose galactose/mannose-N-acetylhexoseamine); Neu5Ac: N-acetylneuraminic acid.

**Figure 4.**
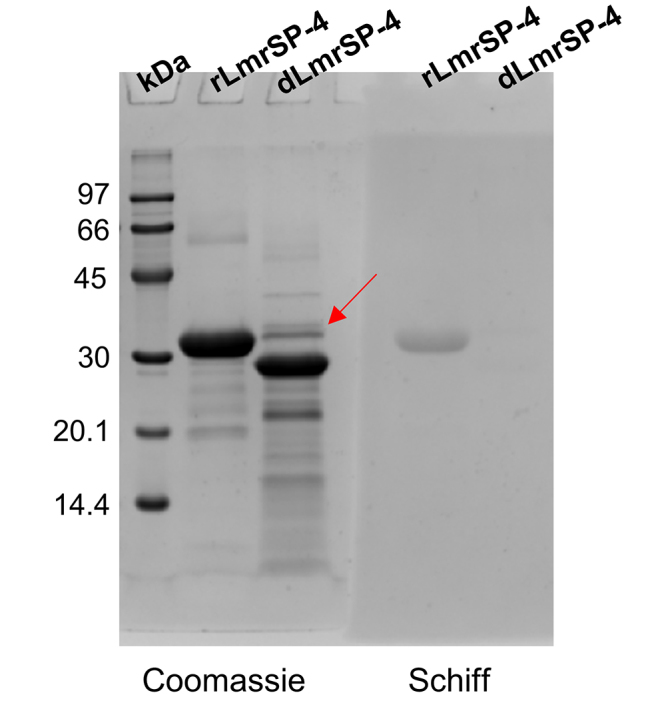
Deglycosylation of LmrSP-4 by PNGase F and visualization of the digested product by 13.5% SDS-PAGE. Gel run was performed at 90 V and gel was divided into two pieces for staining protocol. One of them was stained with *Coomassie Brilliant Blue G-250* ^®^ (blue) and the other one was stained with periodic acid-Schiff (pink for glycoproteins). Picture was acquired in black and white by the Gel Doc™ EZ Gel Documentation System (Bio-Rad, USA). The red arrow indicates PNGase F (36 kDa). Lanes: kDa: molecular weight marker; rLmrSP-4: reduced LmrSP-4; dLmrSP-4: deglycosylated LmrSP-4.

**Figure 5.**
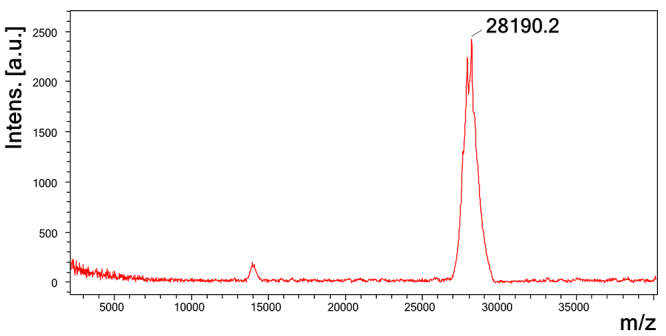
Molecular mass determination of LmrSP-4 through MALDI-TOF mass spectrometry. Sample was diluted in a sinapinic acid matrix and data were acquired in a positive linear mode using the mass spectrometer Ultraflex II MALDI TOF/TOF (Bruker).

LmrSP-4 had its N-terminal determined by Edman degradation up to the 53^rd^ amino acid residue as previously shown (Table 2). We also investigated the internal peptides of this novel serine proteinase to obtain the highest sequence coverage. The primary sequence of LmrSP-4 was verified by digesting the protein with trypsin and Glu-C and peptides were analyzed by MS/MS using two different fragmentation modes (HCD and ETD). Additional file 2 contains all peptides identified in the MS/MS database search (including the false positive ones) and Table 3 shows the correct peptides after manual investigation of the MS/MS search. Multiple sequence alignment of LmrSP-4 with other SVSPs is shown in Fig. 2. In addition, we investigated the occurrence of N-linked sugars. HCD MS/MS spectrum of the ion [M + 4H] ^4+^ = 4971.2538 shows characteristic oxonium ions for N-acetylhexoseamine (HexNAc), N-acetylneuraminic acid (Neu5Ac) and the disaccharide Hex-HexNAc (hexose galactose/mannose linked to a HexNAc), although the peptide containing the glycosylation site could not be identified (Fig. 3). The digestion with PNGase F confirmed that LmrSP-4 is N-linked with a carbohydrate content estimated in 12% and no glycoprotein band was stained after PNGase F treatment (Fig. 4). The molecular mass of LmrSP-4 was determined through MALDI-TOF as 28,190 Da (Fig. 5).

**Table 3.** MS/MS derived sequences after digestion of LmrSP-4 with trypsin and Glu-C used to deduce its primary sequence

**Scan time**	Fragmentation mode	MS/MS derived sequence^*^	z	Observed m/z	Calculated m/z	Mass deviation (ppm)	Score
8.43	HCD	HPCGHPD**G**PGVYTK	3	507.9003	507.9016	-2.56	756.0
9.41	HCD	FICPNR	2	403.7014	403.7025	-2.72	448.4
0.40	HCD	KVPNKDEETRDPKEK	4	453.9911	453.9917	-1.32	539.8
13.92	ETD	VFGGDECNINEHR	3	516.2264	516.2283	-3.68	603.0
19.71	ETD	NDEKDKDIMLIR	3	497.2610	497.2608	0.40	713.0
17.07	ETD	KNDEKDKDIMLIR	4	405.2214	405.2212	0.49	1054.4
21.78	HCD	WVLTAAHCDSE	5	644.7833	644.7850	-2.64	605.8
8.58	ETD	TRDPKEKFICPNRKKNDE	5	455.8381	455.8381	0	825.4
16.24	ETD	NFQMQLGVHSKKVPNKDEE	5	449.6274	449.6274	0	976.9
41.63	ETD	HIALLSLPSSPPSVGSVCRIMGWG**K**ISPTKE	5	661.7568	661.7567	0.15	1232.3
22.68	ETD	KDKDIMLIRLNRPVSNSE	4	536.7926	536.7928	-0.37	1105.4
33.91	ETD	IYPDVPHCADINILD	3	585.6191	581.6188	0.51	444.5
28.31	HCD	HRSLVVLF**D**	2	543.3080	543.3087	-1.28	256.7

^*^ Cys is carbamidomethylated; underlined M is oxidized Met; amino acids in bold are different from the database and were manually confirmed

### Enzyme activities of LmrSP-4

**Figure 6.**
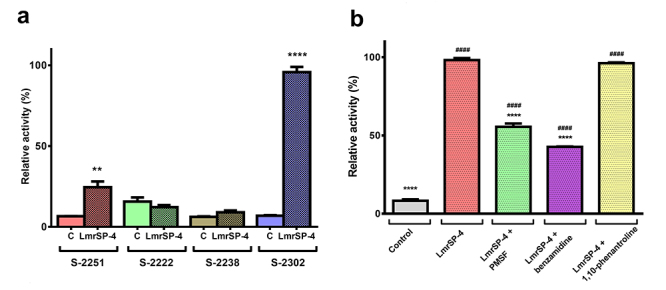
Enzyme activity upon chromogenic substrates. **A:** Enzyme activity was evaluated upon 0.4 mM chromogenic substrates for Factor Xa (S-2222), substrate for thrombin (S-2238), plasmin and streptokinase-activated plasminogen (S-2251) and plasma kallikrein (S-2302), according to the manufacturer’s protocol. Data were analyzed using unpaired t-test and comparing the activity on each substrate tested with its own negative control (** for *p* < 0.01 and **** for *p* < 0.0001). **B:** Enzyme activity of LmrSP-4 was evaluated upon substrate S-2302 in the presence of possible inhibitors (PMSF, benzamidine and 1,10-phenantroline). Data were analyzed by one-way ANOVA followed by Dunnett’s multiple comparisons test (****p < 0.0001 comparing results to LmrSP-4 without inhibitor and ^####^ *p* < 0.0001 compared to the control). Abbreviations: C: control.

LmrSP-4 degrades the fibrinogen Aα-chain as shown in Fig. 1b and 1c, even in the presence of EDTA, but not in the presence of PMSF, evidencing the presence of serine proteinase activity. A densitometry analysis reveals the decreasing of Aα-chain proportion in comparison to the Bβ and γ-chains of fibrinogen. Besides fibrinogen, LmrSP-4 is also active upon the substrate for plasma kallikrein (S-2302) and, in a minor extension, upon the substrate for plasmin and streptokinase-activated plasminogen (S-2251). However, LmrSP-4 has no activity upon the substrate for thrombin (S-2238) nor the substrate for factor Xa (S-2222) (Fig. 6a). Enzyme activity upon S-2302 was also evaluated in the presence of different enzyme inhibitors. The proteolytic activity was decreased by PMSF and benzamidine, but in the presence of 1,10-phenantroline the enzyme remained active (Fig. 6b).

### Determination of the optimal conditions of LmrSP-4

**Figure 7.**
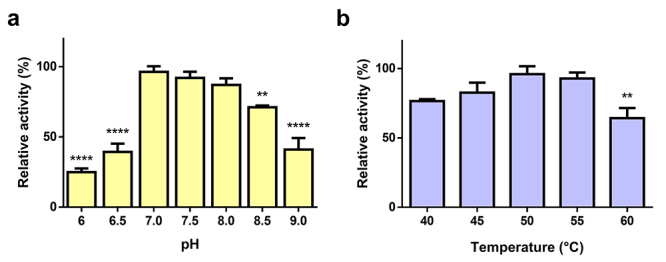
Determination of serine proteinase activity of LmrSP-4 on FRET substrate Abz-KLRSSKQEDDnp. **A:** pH-profile. **B:** temperature-profile. Data were analyzed by one-way ANOVA followed by Dunnett’s multiple comparisons test (**p < 0.01 and ****p < 0.0001). Results were compared to the pH/temperature with the highest activity.

The optimal conditions for LmrSP-4 activity was evaluated at several pHs (6.0-9.0) and temperatures (40-60 °C) using the FRET substrate Abz-KLRSSKQ-EDDnp. LmrSP-4 showed the highest activities within the range from neutral to basic pH values (Fig. 7a) and was very active at high temperatures, starting to denaturate at 60 °C (Fig. 7b).

## Discussion

A transcriptomic study of *L. muta* venom gland [43] revealed major toxin transcripts encode BPPs (73.2%), SVMPs (5.9%), C-type lectin (5.8%), PLA_2_ (4.7%), LAAO (3.7%) and SVSP (3.5%). However, several factors are involved in protein production and proteomic studies from the genus *Lachesis* have shown these venoms are mainly comprised by serine and metalloproteinases (21-35% and 18-38%, respectively), PLA_2_s (2-13%), BPPs (14-28%) and LAAO (0.5-10%) [12, 44, 45]. Despite this, there is a long road ahead to unveil novel components from these venoms. Based on a previous fractionation of LmrV [13], we decided to perform the subproteome of the major fraction from this venom and investigate some of its components.

The S5 fraction mainly presents components of about 30 kDa (with minor components of around 15 kDa) comprising about 26% of the crude soluble LmrV (Fig. 1a-insert; Table 1). This fraction presented proteolytic activity upon fibrinogen (except in the presence of PMSF), indicating the presence of serine proteinases (Fig. 1a-insert). S5 was then submitted to a RP-FPLC using a C4 column and the 6 subfractions (S5C1-S5C6) obtained were analyzed, showing a diverse array of serine proteinases, but also a C-type lectin (Fig. 1b, Table 2).

SVSPs are trypsin-like enzymes since the mammalian trypsin cysteine pattern is conserved in their structure [46]. These proteins are usually related to hemostasis disturbances in snake envenoming and have demonstrated interesting biological activities, including (anti)coagulant action, fibrinolysis, blood pressure reduction and (in)activation of platelet aggregation [47-51].

Concerning the lectin class, there are usually two types of lectins in snake venoms: the sugar binding lectins (or C-type lectins) and the C-type lectin-like proteins (CLPs). The C-type lectins usually show weak toxicity, being the erythrocytes agglutination one of its key roles. In addition, these lectins may contribute to the venom’s antibacterial action during the digestion of preys by recognizing some specific pathogen carbohydrates. On the other hand, CLPs present the carbohydrate recognition domain but lack this activity due to the loss of the calcium binding site, and are usually implicated in anticoagulation effects, platelet activation, as well as antithrombotic action. C-type-lectins and CLPs are reviewed elsewhere [52-55].

S5C6 fraction contains the first lectin described in LmrV, which was named LmrLEC-1. It is only 43% and 38% identical (but 55% and 51% similar) to a C-type lectin from *L. stenophrys* (sp|Q9PSM4) [56] and a C-type lectin precursor from *L. muta* (sp|Q27J51) [43], respectively (Fig. 2c). Lectins already isolated from *Lachesis* venoms are generally dimers of around 28 kDa which are separated under reducing conditions [56, 57]. Therefore, LmrLEC-1 and possibly other lectins in LmrV are likely represented by the low molecular weight band (~14 kDa) in the SDS-PAGE carried out with S5 fraction under reducing conditions (Fig. 1a-insert). The study of snake venom lectins is of utmost importance. It has enabled the discovery of biochemical pathways in homeostasis helping to understand the mechanisms involved in platelet aggregation. Furthermore, they are potential leads for the design of antithrombotic and anticoagulation drugs [53].

Regarding the serine proteinases present in S5 fraction, most of them (LmrSP-2, LmrSP-3 and LmrSP-5) were identified as belonging to the thrombin-like enzymes (TLEs) class. In fact, this class is mainly reported in snakes from Crotalinae subfamily, which comprises the genus *Lachesis* among others [58]. TLEs have been already isolated from *L. muta* venoms [16, 59-61], and two are TLEs from LmrV [23, 60]. The first one was isolated in 1981 when *L. m. rhombeata* was designated as *L. m. noctivaga* [60, 62] and LMR-47 (sp|Q9PRP4) [23] was the only serine proteinase from LmrV whose sequence was available at UniProt so far. A third and last SVSP from LmrV was published in 1985, but it is not a TLE [63].

Furthermore, a vast variety of serine proteinases have been reported in venom proteomes across the genus *Lachesis* [12, 44, 45] but the sequenced amino acid residues presented 100% of identity and unable to identify different isoforms of SVSPs [64, 65]. For example, serine proteinases containing the N-terminal sequence IVGGDECNINEHRFL were identified in the venom proteomes of *L. muta* [45], *L. stenophrys* [45] and *L. melanocephala* [44], and the same sequence was determined in fractions S5C2 and S5C3 not allowing differentiating the present enzymes. These enzymes may be result of amino acid mutations, post-translational modifications (e. g. glycosylation) or other modifications (e. g. metoxilation) that lead to different interactions with the chromatographic resin and, consequently, different elution times.

In this study, the SVSPs identified were denominated from the number 2 (e. g. LmrSP-2) since they will be the second, third, fourth and fifth sequences from LmrV available at UniProt database. The accession numbers are listed on Table 2. Sequences of LmrSP-2 and LmrSP-3, represented by fractions S5C1 and S5C2, respectively, resemble those of other serine proteinases from *L. muta* subspecies. The presence of distinct serine proteinases within snake venoms may lead to different substrate specificities and functional diversity in these venoms. The first 20 amino acid residues of LmrSP-2 are 100% identical to a TLE isolated from *L. m. muta* [66] while LmrSP-3 is similar to a kallikrein-like enzyme from *L. m. rhombeata* [24]. However, they are not the same enzymes as those already published and represent a novelty in LmrV. The TLE isolated from *L. m. muta* has more than 40 kDa [66] and this molecular mass was not detected in our analysis (Fig. 1a-insert) although it is ~10% of S5 fraction, and LmrSP-3 has a Leu residue in the 10^th^ position while the kallikrein-like enzyme from *L. m. rhombeata* presents a Leu [24].

Concerning LmrSP-5 present in the S5C5 fraction, the first 25 amino acid residues are identical to LmrSP-4. The enzymes are assumed to be different since they are eluting at different points in the gradient (Fig. 1b), but it was not possible to differentiate them solely based on the N-terminal sequencing. LmrSP-4 sequence was chosen for database submission since it is the longest one. The first 13 N-terminal amino acid residues (VFGGDECNINEHR) from LmrSP-4 have been already detected in proteomic analyses of *L. muta* [45], *L. m. rhombeata* [12] and *L. stenophrys* venoms [44], but this sequence obtained in these studies was too short that the identification of specific isoforms was impossible. The N-terminal from LmrSP-4 shared the highest identity with LV-PA (EC 3.4.21), a plasminogen activator from *L. m. muta* (sp|Q27J47), whose complete amino acid sequence was obtained through molecular cloning [67]. The N-terminal fragment determined by Edman degradation revealed differences in the positions 20 and 22 in comparison to LV-PA. LV-PA presents Asn20 and Ser22 whilst both positions are represented by Asp residue in LmrSP-4. Asn20 is the unique N-glycosylation in LV-PA [67], therefore its change by an Asp residue leads to the loss of a potential N-glycosylation site in LmrSP-4. Other differences between them are at positions 125 (T-K) and 206 (E-G). LV-PA and LmrSP-4 share 97.5% of identity and 98.1% of similarity taking into account only the aligned residues between them.

LmrSP-4 is also very similar to TSV-PA from *Trimeresurus stejnegeri* [68], sharing 87.5% of identity in the aligned amino acid residues. Interesting modification is found in the region 80-82 (numbering in this study), specifically in the position 80, where there is a replacement of an Asp by an Asn. Implications of this modification will be discussed further. Additionally, LmrSP-4 is structurally very similar (64 to 74%) to snake venom kallikrein-like enzymes (Fig. 2b), which are intrinsically responsible for causing hypotension in preys [14, 49].

LmrSP-4 showed reduction of about 4 kDa, corresponding to 12% (m/m) of its molecular mass estimated by SDS-PAGE, after PNGase F treatment. LmrSP-4 was stained by Schiff reagent only before PNGase F treatment, indicating all carbohydrate content is N-linked and was removed by PNGase F. While plasminogen activators usually present one N-glycosylation site in the N-terminal region (position 20^th^, Fig. 2a), LmrSP-4 present a substitution in this position leading to the loss of this N-glycan site. MS/MS analysis revealed the presence of glycopeptides, but the glycan site could not be determined. Fig 3 shows the carbohydrate marker ions HexNAc, Hex-HexNAc and Neu5Ac. Considering that N-linked carbohydrate are linked to an Asn residues in the consensus sequence Asn-X-Ser/Thr, where X can be any amino acid except Pro [69], the LmrSP-4 N-glycan site is likely present in the region 161-163 for which there was no peptide sequence coverage by MS/MS (Fig. 2a).

The degree of glycosylation varies among SVSPs and this post-translational modification seems to be related to the macromolecular selectivity and unusual thermal stability that these enzymes may present [70, 71]. TLEs are usually glycosylated in a major extent than plasminogen activators or kallikrein-like enzymes. SVSPs with plasminogen-activating or kallikrein-like activity from the genus *Lachesis* present molecular mass within the range of 27.9 to 33 kDa estimated by SDS-PAGE [14, 24, 67, 72]. On the other hand, *Lachesis* TLEs are usually larger proteins of more than 40 kDa [16, 23, 73]. However, after deglycosylation reaction, all of them present 27-28 kDa [67, 72, 73]. Glycosylation is also responsible for heterogeneity among SVSPs. TLE-B and TLE-P from *L. m. muta* present very similar structural, functional and immunological properties but different glycosylation degrees [73]. In addition, stenoxobin from *L. stenophrys* [59] and a kininogenin from *Vipera ammodytes ammodytes* [74] has heterogeneity due to sialic acid. Therefore, glycosylation is a post-translational modification intrinsically related to the presence of serine proteinases isoforms in snake venoms and might explain the same N-terminal sequences for S5C2 and S5C3 (Table 2). However, other assays are necessary to further characterize these proteins. The molecular mass of LmrSP-4, determined by MALDI-TOF as 28,190 Da, is in accordance with the mass estimated by SDS-PAGE and with the mass of other SVSPs as discussed above.

Although SVSPs are very similar in terms of primary structure, post-translational modifications and/or surface residues may exert important role in substrate specificity. Partially deglycosylated proteinases from Russell’s viper venom have shown lower fibrinogenolytic activity than the native enzymes [75]. Another example includes the key residue Asp80 (our numbering) in TSV-PA. The peptide KKDDEV (amino acid residues 78-83) is close to the catalytic site in the three-dimensional structure and the residue Asp80 takes part in electrostatically interactions between TSV-PA and plasminogen. The replacement of Asp80 by an Asn residue resulted in lower activity upon plasminogen [68]. LmrSP-4 naturally presents the residue Asn80, which explains its lower activity upon the substrate S-2251.

In comparison to TLEs, plasminogen activators comprehend a small number of proteins among the snake venom serine proteinases [46]. LV-PA from *L. m. muta*, is an acid glycoprotein active upon S-2251 and Tos-Gly-Pro-Lys-pNA (another substrate for plasmin) and cleaves fibrinogen but does not induce blood coagulation [15].

LmrSP-4 has an outstanding activity upon the substrate for plasma kallikrein (S-2302) but was not active upon S-2222 and S-2238, substrates for factor Xa and thrombin, respectively. Kallikreins are trypsin-like proteins and thus are able to hydrolyze different substrates, although their specificities are not as broad as trypsin [76]. The first kallikrein-like protein from *Lachesis* was isolated from its venom more than 20 years ago and released bradykinin (BK) from bovine kininogen [14]. LV-Ka is another kallikrein-like serine proteinase isolated from *Lachesis muta* whose structure and function was extensively studied [49, 72]. This enzyme is active against substrates for plasma and glandular kallikrein and plasmin [49, 72], releases BK from bovine fibrinogen [72] and decreases blood pressure in rats [49].

Enzyme activity upon substrate S-2302 in the presence of possible inhibitors confirmed that LmrSP-4 is a serine proteinase. Proteolytic activity decreased in the presence of PMSF and benzamidine. Both are serine proteinase inhibitors but differ in their inhibition mechanism. While PMSF reacts with catalytic Ser residues in these proteins [77], benzamidine is a reversible competitive inhibitor [78]. On the other hand, 1,10-phenantroline is a metalloproteinase inhibitor which competes by the zinc ions needed for the proteinase activity [79]. Herein, this inhibitor did not affect the LmrSP-4 proteolytic activity, indicating that zinc ions are not essential for the catalytic activity. However, it is interesting that zinc ions are structurally relevant for ABUSV-PA, a plasminogen activator from *Agkistrodon blomhoffii* Ussurensis venom, although they do not influence its activity [80].

LmrSP-4 consumes fibrinogen Aα-chain (Fig. 1b and 1c) except in the presence of PMSF, a serine proteinase inhibitor. The proportion among the Aα, Bβ and γ chains remains the same comparing fibrinogen chains after its treatment with LmrSP-4 pre-incubated with PMSF (Fig. 1c). Besides the Aα consumption, there is a difference in the migration pattern close to the Aα chains (Fig. 1c), which might indicate the release of fibrinopeptide A from the Aα chain. However, a more specific assay is needed to confirm this hypothesis. TLEs from *Lachesis* usually release fibrinopeptide A but some of them also slowly split off fibrinopeptide B from fibrinogen [16, 23, 61, 73]. During the envenoming process, thrombin-like serine proteinases act on fibrinogen converting that in a different form of fibrin which does not form a solid fibrin clot. As time goes by, the fibrinogen of prey/victim is all consumed, and blood becomes uncoagulable [81]. On the other hand, the action of TLEs upon fibrinogen has enable the production of the fibrin sealant: a mixture of fibrinogen-rich cryoprecipitate from *Bubalus bubalis* blood and a TLE from *Crotalus durissus terrificus* snake venom, which is in clinical trial for the treatment of chronic venous ulcers and has shown promising results as adjuvant in the peripheral nerve injury treatment [82-84].

Regarding optimal conditions for LmrSP-4 enzyme activity, we firstly evaluated the pH condition incubating enzyme and FRET substrate in different pH values at 40 °C, a medium temperature for enzymes that act in physiological conditions. LmrSP-4 showed highest substrate hydrolysis in pH 7 although there was no statistically significant difference in activity within the pH range from 7 to 8 (Fig. 7a). The optimum pH determined for LmrSP-4 is in accordance to other SVSPs, especially those from *Lachesis* venoms [16, 23, 60, 61]. Temperature influence was evaluated at pH 7 and the enzyme showed the highest activity at 50 °C but there was no statistically significant difference in activity at 40, 45 and 55 ºC (Fig. 7b). Substrate hydrolysis decreased at 60 °C showing the beginning of a denaturation process and loss of activity. SVSPs are usually resistant to high temperatures [85-87] and this may be due to their sugar content and three-dimensional structure stabilized by the six conserved disulfide bonds [70].

## Conclusions

Analysis of the dominant fraction of LmrV after molecular exclusion chromatography revealed the presence of novel and different serine proteinase isoforms in this venom as well as the first lectin from LmrV. A kallikrein-like serine proteinase (LmrSP-4) that might be useful as molecular tool for investigating bradykinin-involving process was isolated and biochemically characterized.

## Abbreviations

 ACN: acetonitrile; AGC: automatic gain control; AMBIC: ammonium bicarbonate; BK: bradykinin; BPPs: bradykinin potentiating peptides; CLP: C-type lectin-like protein; CRISP: cysteine-rich secretory protein; DTT: 1,4-dithiothreitol; EDTA: ethylenediamine tetraacetic acid; ETD: electron transfer dissociation; FRET: fluorescence resonance energy transfer; HCD: higher-energy collisional dissociation; HexNAc: N-acetylhexoseamine; LAAO: L-amino acid oxidase; Lmr: *Lachesis muta rhombeata*; LmrV: *Lachesis muta rhombeata* venom; MALDI-TOF MS: matrix-assisted laser desorption/ionization time-of-flight mass-spectrometry; Neu5Ac: N-acetylneuraminic acid; PAGE: polyacrylamide gel electrophoresis; PLA_2_: phospholipase A_2_; PMSF: phenylmethylsulfonyl fluoride; RP-FPLC: reversed-phase fast protein liquid chromatography; SDS: sodium dodecylsulphate; SVSP: snake venom serine proteinase; TFA: trifluoroacetic acid; TLEs: thrombin-like enzymes; VEGF: vascular endothelial growth factor.

## Supplementary material

TThe following online material is available for this article

Additional File 1: Each spreadsheet contains the first 100 results from the blastp search performed for the N-terminal sequence from each reversed-phase chromatography fraction (S5C1-S5C6).

Additional File 2: Table containing all MS/MS-derived sequences after the digestion of LmrSP-4 with trypsin and Glu-C.
